# Diffuse Systemic Sclerosis with Left Ventricular Diastolic Dysfunction: A Case Report

**DOI:** 10.31729/jnma.4768

**Published:** 2019-12-31

**Authors:** Asmita Neupane, Prabesh Luintel, Subodh Sagar Dhakal

**Affiliations:** 1 Kathmandu Medical College and Teaching Hospital, Sinamangal, Kathmandu, Nepal; 2 Department of Internal Medicine, Kathmandu Medical College and Teaching Hospital, Sinamangal, Kathmandu, Nepal

**Keywords:** *connective tissue disease*, *immune-mediated*, *rheumatic disease*, *scleroderma*, *systemic sclerosis*

## Abstract

Systemic sclerosis is a connective tissue disease characterized by wide-spread vascular lesions and fibrosis of the skin and internal organs. It is an immune mediated rheumatic disease with the presence of an immunological dysfunction of T lymphocytes, especially Th1 and Th17 subtypes. It affects gastrointestinal, pulmonary, vascular, musculoskeletal, cardiac and various other systems. This disease is rare but has high morbidity and mortality with less known effective management. We report a case of 70-year-old female with systemic sclerosis presented with pain along with swelling over multiple joints since 18 months which exacerbated since last 6 months and wound over finger tips since last 2 weeks. We present here other various signs, investigations and management of this uncommon disease systemic sclerosis, also known as scleroderma. Various systems are evident to be involved including cardiac (left ventricular diastolic dysfunction) and peripheral vascular system (Raynaud's phenomenon).

## INTRODUCTION

Systemic Sclerosis (SSc) is a connective tissue disease characterized by diffuse vascular lesions and fibrosis of the skin and involvement of major organs including lungs, kidneys and heart. Primary myocardial involvement is common in SSc and when clinically evident, appears as a poor prognostic factor and leading cause of mortality.^1^ Although systemic sclerosis is uncommon, it has a high morbidity and mortality. Improved understanding of systemic sclerosis has allowed better management of the disease, including improved classification and more systematic assessment and follow-up.^2^ Our report presents the case of systemic sclerosis with left ventricular diastolic dysfunction.

## CASE REPORT

A 70-years-old female presented and was admitted on our hospital for chief complaints of pain along with swelling over multiple joints since 18 months which exacerbated since last 6 months and wound over finger tips since last 2 weeks. The joint pain was insidious in onset, simultaneously affecting both the small joints: wrist, interphalangeal joints and large joints: knee, elbow, shoulder and ankle. Joint pain was bilateral, constant, non-radiating, associated with reduced mobility especially at the beginning of motion, associated with early morning stiffness and relieved on taking medication. She also complained of bluish discoloration of fingers on exposure to cold that painfully changed to red after warming up. She reported that her skin progressively tightened up since last 2 months with reduced sweating. She also gave complain of wounds over her finger tips that were spontaneous with no history of trauma.

**Figure 1:**
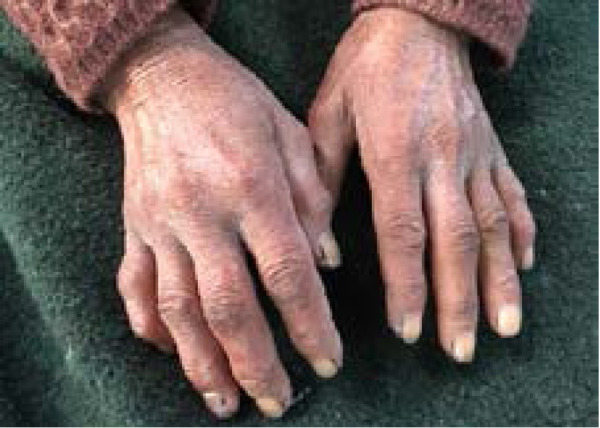
Dorsum of hand showing dry, lustrous and glabrous skin with reduced creases and flexion deformity with swollen proximal and distal interphalangeal joints.

**Figure 2:**
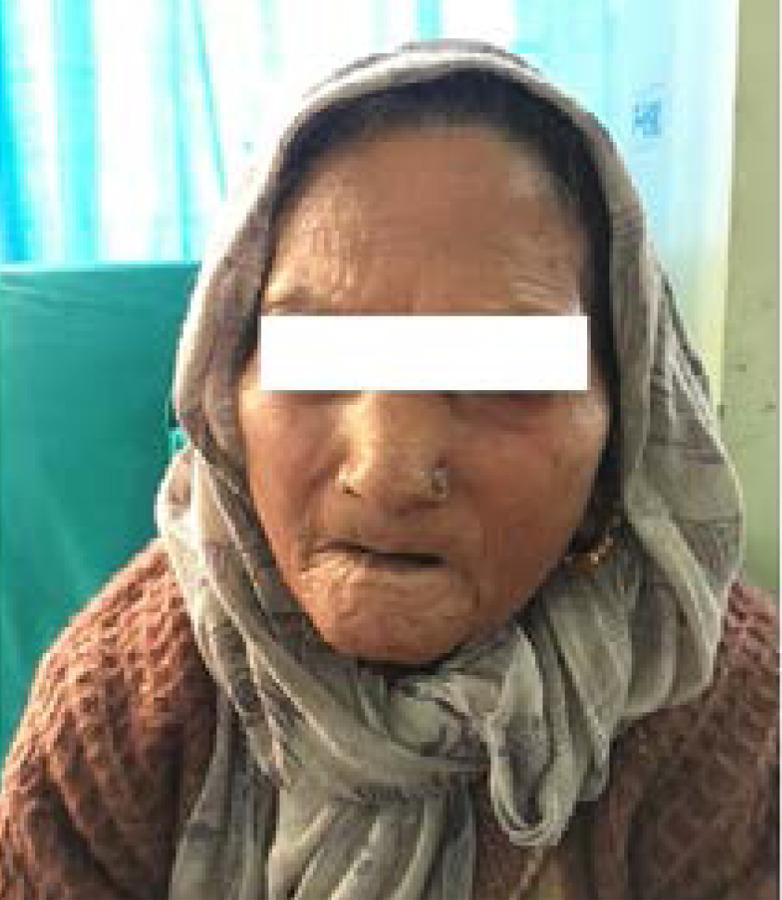
Mask-like face with dry lustrous skin, reduced wrinkles, thin lips, perioral radial grooves and microstomia.

On general examination and dermatological examinations, she had dry, lustrous and glabrous skin with reduced transverse creases over the dorsum of fingers (Figure 1). Flexure contracture of involved joints with limited range of motion was present. Face had taut and shiny skin with loss of wrinkles and expressionless face. Lips were thin with radial furrowing around mouth. Microstomia with two-finger-breath mouth opening and pinched beak-like nose were apparent (Figure 2). Nail pitting and bilateral pedal edema were present. All of her vitals were stable. Chest examination was normal. Cardiovascular examination revealed normal heart sounds with no murmur. Abdomen was soft, nontender with bowel sound present and no organomegaly. Central nervous system was grossly intact.

On investigations, her hemoglobin was 10.3mg/dL, total white blood cell count was 13,800cells/mm3 with 76% Neutrophils and 28% Lymphocytes. Platelets were 1,75,000/mm3, Fasting Blood Glucose was 65mg/dL, serum sodium concentration was 145meQ/dL and serum potassium was 3.1meQ/dL. Her C-Reactive Protein (CRP) was negative. Creatinine Phosphokinase-Muscle/Brain(CPK-MB) was 24IU/L and Troponin-I was negative. Her Antinuclear Antibody (ANA) titre was 3.76 (positive) and Anti-Scl-70/Anti-topoisomerase-I (3.82/++) were positive, Erythrocyte Sedimentation Rate (ESR) was 32mm/hr. Echocardiography performed by tissue Doppler method showed left ventricular diastolic dysfunction and mild pericardial effusion. Pulmonary Function Tests couldnot be done because of her microstomia.

On the 3^rd^ day of her hospital stay, she developed loose stools which was managed with oral rehydration, zinc tablets and probiotics. Based on clinical findings and rheumatic panel report, oral prednisolone and hydrocortisone injection therapy was initiated along with calcium and vitamin D supplements. Mupirocin ointment was applied on the skin ulcers over fingers. After a week of hospital admission, she was asked to come to follow up in the hospital and also referred to a rheumatologist for further management.

## DISCUSSION

Systemic sclerosis, also called scleroderma, is an immune-mediated rheumatic disease that is characterised by fibrosis of the skin and internal organs and vasculopathy.^2^

It is due to vascular and immune dysfunction, the pathogenesis of which is complex and poorly understood.^3^ Some risk factors are known and include combination of persistent Raynaud's phenomenon, steroid hormone imbalance, selected chemicals, thermal, or other injuries.^4^ A complex autoimmune response, involving innate and adaptive immunity with specific/functional autoantibody production, characterizes the disease.^4^ Progenitor circulating cells (monocytes, fibrocytes), together with growth factors and cytokines participate in disease diffusion and evolution.^4^

Autoantibodies against topoisomerase I (Topo I, ATA, Scl-70) are observed with antinuclear antibodies (ANA) being the most frequently detected autoantibodies.^5^ In our case, the investigated antinuclear autoantibodies and Scl-70 were found to be positive.

Systemic sclerosis is classified into two subsets based on the extent of skin involvement-limited systemic sclerosis (lcSSc) and diffuse systemic sclerosis (dsSSc). Patients with fibrosis of the skin affecting acral parts of the body, face and limbs (distal to the knees and elbows) are classified as having lcSSc, whereas those with fibrosis of the trunk and proximal parts of the limbs are classified as having dsSSc.^5^

Skin involvement includes skin thickening, Raynaud's phenomenon, hypo or hyperpigmentation, telangeictasia, mask-like face, radial furrowing around mouth, narrowed mouth (microstomy), swollen (puffy fingers), sclerodactyly (diseased finger contracture); GI involvement includes dysphagia, odynophagia, regurgitation; Musculoskeletal involvement manifests with restricted joint mobility, joint contractures, joint stiffness, mainly in metacarpo-phalangeal joints, wrists, knee, distal and proximal interpahalangeal joints, joint pain, acro osteloysis (resorption of terminal phalanges), muscle weakness and pain; Cardiac involvement includes myocardial fibrosis, myositis, coronary heart disease, right and left heart failure, arrythmia, valvular complications, systolic and diastolic dysfunction.^5^ Presentation with primary myocardial disease, i.e. without systemic or pulmonary hypertension and without significant pulmonary or renal disease is common.^6^ Reduced RVEF appears to be a common feature in early SSc.^7^

The case presented has features of diffuse systemic sclerosis, i.e. dry, lustrous, glabrous skin with reduced transverse creases over the dorsum of fingers, flexure contracture of involved joints with limited range of motion, face with taut and shiny skin with loss of wrinkles and mask-like face, thin lips with radial furrowing around mouth, microstomia, pinched beaklike nose, nail pitting, raynaud's phenomenon, wound over the terminal phalanges, multiple joint pain with swelling of joints of hands.

## References

[1] Kahan A, Allanore Y (2006). Primary myocardial involvement in systemic sclerosis. Rheumatol.

[2] Denton CP, Khanna D (2017). Systemic sclerosis. Lancet.

[3] Katsumoto TR, Whitfield ML, Connolly MK (2011). The pathogenesis of systemic sclerosis. Annu Rev Pathol.

[4] Cutolo M, Soldano S, Smith V (2019). Pathophysiology of systemic sclerosis: current understanding and new insights. Expert Rev Clin Immunol.

[5] Sobolewski P, Maślińska M, Wieczorek M, Łagun Z, Malewska A, Roszkiewicz M (2019). Systemic sclerosis-multidisciplinary disease: clinical features and treatment. Reumatologia.

[6] Kahan A, Coghlan G, McLaughlin V (2009). Cardiac complications of systemic sclerosis. Rheumatol.

[7] Meune C, Allanore Y, Devaux J, Dessault O, Duboc D, Weber S (2004). High Prevalence of Right Ventricular Systolic Dysfunction in Early Systemic Sclerosis. J Rheumatol.

